# Uterine Radial Artery Resistance Index Predicts Reproductive Outcome in Women with Recurrent Pregnancy Losses and Thrombophilia

**DOI:** 10.1155/2019/8787010

**Published:** 2019-06-20

**Authors:** S. H. Bao, N. Chigirin, V. Hoch, H. Ahmed, S. T. Frempong, M. Zhang, J. L. Ruan, J. Kwak-Kim

**Affiliations:** ^1^Reproductive Medicine and Immunology, Department of Obstetrics and Gynecology, Department of Microbiology and Immunology, Chicago Medical School, Rosalind Franklin University of Medicine and Science, Vernon Hills, IL 60061, USA; ^2^Department of Reproductive Immunology, Shanghai First Maternity and Infant Hospital, Tongji University School of Medicine, Shanghai 201204, China

## Abstract

Uterine radial artery resistance index (URa-RI) by Doppler ultrasound may reflect the changes in the uteroplacental circulation and be associated with adverse events in early pregnancy. Recurrent pregnancy losses (RPL) are associated with thrombophilia, and anticoagulation treatment with low molecular weight heparin improves pregnancy outcome in women with RPL and thrombophilia. A retrospective cohort study was conducted in 139 pregnant women with 3 or more RPL and thrombophilia. The relationship between pregnancy outcome and dynamic changes of URa-RI was analyzed in 116 women who delivered a liveborn infant and 23 who miscarried the index pregnancy. Patients were on preconception low molecular weight heparin, low-dose aspirin (81mg per day), and prednisone treatment. URa-RI was measured during periovulation time, at the time of positive pregnancy test, and then repeated every two weeks until 32-week gestation or the time of miscarriage. The URa-RI at 8-week gestation was significantly higher in women who miscarried the index pregnancy than those who delivered alive born infant (0.51±0.08 vs. 0.42±0.03, P<0.001). Receiver operating characteristic curve analysis demonstrated that URa-RI of 8 wk gestation effectively distinguished women who miscarried from those who had a live birth with an area under the curve of 82.6% (95% CI 69.01-97.17). After adjusting for covariates including age, BMI, and number of miscarriages, multiple logistic regression models showed that each 0.1 unit increase of URa-RI of 8 wk gestation was associated with 18.70-point increase in the risk of miscarriage (OR19.70, 95%CI 4.26-91.1, P<0.001), and women with an URa-RI≥0.45 had an OR of 49.48 (95% CI 8.01-307.95; P<0.001) for miscarriage compared to those who had URa-RI<0.45. In women with RPL and inherited thrombophilia, increased URa-RI at 8-week gestation was associated with spontaneous abortion independent of other risk factors while they were on anticoagulation treatment.

## 1. Introduction

Recurrent pregnancy loss (RPL) is a common reproductive health problem, which affects 2-5% of couples in reproductive age. RPL is defined as two or more clinical pregnancy losses which are documented by ultrasonography or histopathological examination by American Society for Reproductive Medicine [1]. Reflecting the physical, emotional, and social distress of the affected couples, this updated definition has been rapidly adopted in recent years, including the European Society for Human Reproduction and Embryology [2]. The incidence of RPL has been significantly increased in the recent 10 years, which may suggest its association with environmental changes, to which immune and inflammatory responses are particularly sensitive [3]. With the clinically available test and efforts undertaken to define possible causes for RPL, up to 50% of the cases remain without a clear etiology [1, 4, 5]. Among those women with unexplained RPL, thrombophilia and immune-inflammatory abnormalities have been reported [6].

Inherited thrombophilia, which is a genetic condition that increases the risk of thromboembolic disease, has been reported to be associated with RPL in the last decades [7–13]. Other prospective studies have reported contradictory results, although retrospective studies suggested a modest link between Factor V Leiden gene heterozygosity (and possibly prothrombin gene heterozygosity and protein C and S deficiency) and fetal loss after 10 weeks and particularly for none recurrent loss after 20 weeks [7, 12, 14–18]. This suggests that association is modest and primarily limited to high-risk populations. During pregnancy, the thrombogenic potential of these inherited disorders is enhanced because of the hypercoagulable state by pregnancy-associated changes in the various coagulation factors [19]. Hence, testing for inherited thrombophilia, such as Factor V Leiden, prothrombin gene G20210A and MTHFR C677T polymorphisms, protein C and S deficiencies, and anti-thrombin antibodies, has been recommended, if there is a history of RPL, second or 3rd-trimester obstetrical complications, or a personal or a strong family history of thrombosis. 

The changes in the uterine artery before and during pregnancy reflect the perpetual growth and development of the uteroplacental circulation. Pulsed Doppler ultrasonography of the uterine arteries has been reported to be a useful tool for identifying impaired uterine circulation in women with unexplained RPL [20]. Uterine artery blood flow increases progressively during the luteal phase, with the highest flow encountered in the period that temporally coincides with the implantation window [21]. Recent studies demonstrated that uterine artery blood flow, as measured by uterine artery resistance index (RI), remains at a constant level until the 10th week of pregnancy. Contrarily, uterine radial artery RI (URa-RI) sharply decreases after 5 weeks of pregnancy, suggesting that, in early pregnancy, the uterine radial artery may reflect blood supply to a fetus more accurately than uterine artery [22]. However, studies demonstrating the changes in URa-RI are limited in pregnant women with thrombophilia and anticoagulation treatment. Uterine blood flow was reported to be associated with increased peripheral blood natural killer (NK) cell levels, and low molecular weight heparin (LMWH) treatment improved uterine blood flow in women with unexplained RPL (uRPL) and decreased uterine blood flow [23].

Thus, in this study, we aim to investigate if blood flow measurements by Doppler ultrasound of the uterine radial artery during early pregnancy predict the adverse pregnancy outcome in women with URPL and inherited thrombophilia.

## 2. Materials and Methods

### 2.1. Study Participants

**Figure 1:**
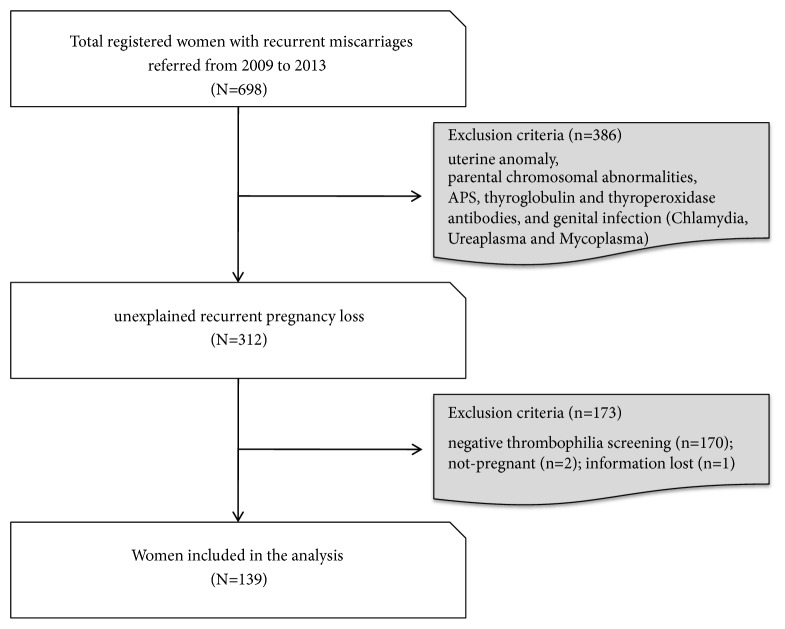
The study flow chart.

We carried out a retrospective cohort study at the Reproductive Medicine, Department of Obstetrics and Gynecology, Chicago Medical School, Rosalind Franklin University of Medicine and Science. The study was approved by the Institutional Review Board of Rosalind Franklin University of Medicine and Science. The individual consent process was waved by the IRB. The study flow is presented in Figure 1. A total of 698 women with 3 or more RPL were registered at the Reproductive Medicine from December 2009 to December 2013. Out of 698 women, 386 women with known etiologies for RPL, such as anatomical anomalies of the genitourinary tract, parental chromosomal aberrations, chronic medical conditions, infections, or hormonal disturbances, were excluded from the study. All the study populations were tested for autoantibodies, such as antiphospholipid antibody (APA), anti-nuclear antibody (ANA), autoantibodies to dsDNA, ssDNA, histone, and Scl-70, anti-thyroperoxidase antibody, anti-thyroglobulin antibody, and thrombophilia as listed in Table 1.

Among 312 women with RPL, women without inherited thrombophilia (n=170), inability to conceive after referral (n=2), and missing follow-up (n=1) were excluded from the study, and a total of 139 women with RPL and thrombophilia were included in the analysis (Figure 1). None had a history of clinical thrombotic events. All received anti-inflammatory (prednisone and/or intravenous immunoglobulin G (IVIg)) and anticoagulant (low molecular weight heparin (LMWH) and low-dose aspirin (LDA)) therapy before conception as previously reported [24].

### 2.2. Evaluation of Uterine Blood Flow

**Figure 2:**
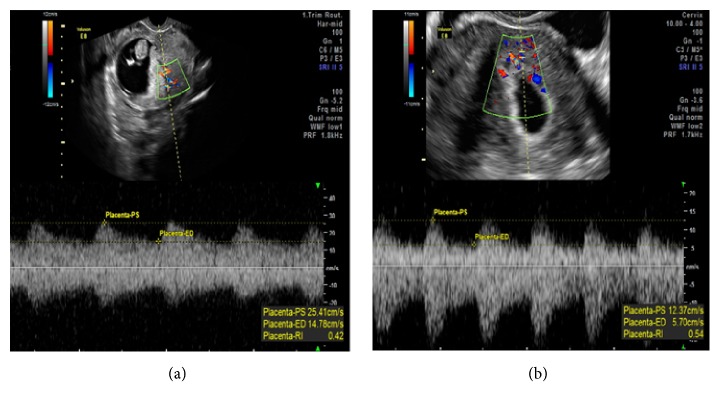
The uterine radial artery resistance index (URa-RI) was monitored with a Doppler ultrasound; (a) URa-RI women who delivered a liveborn infant and (b) URa-RI of women who miscarried the index pregnancy.

To obtain URa-RI, an ultrasonographic scan of the uterus was performed. The midsagittal section of the uterus was secured, and the radial arteries were identified in the mid-section of the myometrium as previously reported [23]. The URa-RI at the ascending branch of the artery was measured with Voluson 730 Expert (version 5.0x; GE Medical Systems). The URa-RI was monitored with an ultrasound at the time of a positive pregnancy test and every two weeks until 32-week gestation or the time of miscarriage (Figures 2(a) and 2(b)). During clinical follow-up, it was inevitable that some subjects did not follow the prescribed time to visit the clinic. We “pooled” the 4- and 5-week data and present as 4-week data. This method was applied to the subsequent weeks of pregnancy. In summary, we analyzed and compared the URa-RI levels before pregnancy (baseline) and at 4, 6, 8, 10, 12, 14, 16, 18, 20, 22, 24, 26, 28, 30, and 32 weeks during pregnancy to predict pregnancy outcomes.

### 2.3. Inherited Thrombophilia Screen

All participants had acquired and inherited thrombophilia workup [24] including protein C and S, plasminogen activator inhibitor-1, and total homocysteine (tHcy) measured by ACL TOP automated coagulometer (Instrumentation Laboratory Co., Lexington, MA, USA). Factor V Leiden R506Q, PAI-1 4G/5G, HPA-1a (HPA-1a/1b), *β*-fibrinogen 455G/A, Factor-II G20210A, Factor XIII VAL34Leu, MTHFR C677T, and A1298C polymorphisms were investigated using DNA samples by real-time polymerase chain reaction (RT-PCR) using a commercial kit (Light Cycler 2.0, Roche Diagnostics) according to the manufacturer's instructions.

### 2.4. NK Cell Fraction, NK Cell Cytotoxicity, and Th1/Th2 Cells Study

Peripheral blood CD56^+^ NK cell proportion, NK cell cytotoxicity (NKC), and Th1/Th2 cell ratios were measured by flow cytometry before pregnancy as previously reported [25, 26]. Briefly, venous blood was drawn from the patient (10 mL). NK cells were determined by detecting the CD3-CD56+ NK cells. After flow cytometric analysis, 12% of peripheral blood NK (pNK) cell fraction among the lymphocytes is defined as the cut-off levels to define a high-risk population of RPL. NKC was measured by using K562 cells as target cells at an effector to target cell (E:T) ratio of 50:1, 25:1, and 12.5:1 as previously reported [27]. Th1 and Th2 cells were determined by detecting the intracellular tumor necrosis factor (TNF)-*α*, interferon (IFN)-*γ*, and IL-10 production. Th1/Th2 cell ratios were calculated as TNF-*α*/IL-10 and IFN-g/IL-10 producing CD4+ cell ratios [25].

### 2.5. Autoimmune Evaluation

Screening for anti-nuclear antibodies (ANA) was performed by indirect immunofluorescence using a commercially available kit (Immunoconcepts, Sacramento, CA, USA). Individual autoantibodies to dsDNA, ssDNA, histone, Scl-70, thyroglobulin, and thyroperoxidase (TPO) were tested by ELISA using corresponding commercially available kits (Inova Diagnostic, San Diego, CA, USA). Antiphospholipid antibodies were tested by enzyme-linked immunosorbent assay (ELISA) [27].

### 2.6. Statistical Analysis

Statistical analysis was performed with R (http://www.R-project.org) and EmpowerStats software (www.empowerstats.com, X&Y solutions, Boston, MA, USA). Comparisons between the live birth and miscarriage groups were performed using *χ*^2^ analysis for categorical variables and two-sample t-test for continuous variables. A receiver operating characteristic (ROC) curve was generated and a cut-off value of URa-RI at 8-week gestation for predicting upcoming miscarriage was determined. A multivariate logistic regression model was fitted while adjusting for age, body mass index (BMI), and the number of previous miscarriages, and adjusted odds ratio (OR) and 95% confidence interval (CI) were calculated. A power simulation model was used to determine the effect of sample size on power. Then the power for different sample size was evaluated (Supplement [Supplementary-material supplementary-material-1]). When probability values are less than 0.05, it is considered to be statistically significant. Data are presented as mean ± SD or proportions.

## 3. Results

### 3.1. Characteristics of the Study Population

Table 1 shows the maternal characteristics, including age, BMI, number of miscarriages, and auto- and cellular immunity of the study group before the index pregnancy. The mean and SD of the age was 34.9 ± 4.5. Twenty-five (25) of the subjects (18.0%) were 40 years or older, and 114 (82.0%) were younger than 40 years. BMI of the study group was 25.2 ± 4.7 Kg/m^2^. Sixty-one of the women (43.9%) were overweight or obese (BMI> 25 Kg/m^2^), 76 (54.7%) had BMI less than 25, and 2 (1.44%) had BMI less than 19. Furthermore, 32 women had 5 or more miscarriages (23.0%). Of the 139 study subjects, 77 were ANA positive (55.4%), and 70 were APA positive (50.4%). Noticeably, 22.5% of the study population had elevated peripheral blood CD56^+^ NK cells (>12%). The mean NK cytotoxicities at E:T ratio of 50:1, 25:1 and 12.5:1 were 16.35 ± 6.5%, 11.02 ± 5.31, and 7.05 ± 4.19%. In regard to Th1/Th2 cell ratios, 52.7% had increased TNF-*α*/IL-10 producing Th cell ratio and 22.6% had increased IFN-*γ*/IL-10 Th cell ratios.

Women who miscarried had significantly higher NK cytotoxicities before the index pregnancy at E:T ratio of 50:1 and 25:1 as compared with those of women who delivered a liveborn infant (P<0.05, respectively) (Table 1). The other clinical characteristics mentioned above were not different in women who miscarried as compared with those of women who delivered a live born infant.

The phenotypic expression of hereditary thrombophilia is listed in Table 2. MTHFR and PAI-1 gene polymorphism were the two most frequent gene mutations among others. Contrarily, Factor-II polymorphism and protein C deficiency were only one and two cases respectively.

### 3.2. Dynamic Changes of URa-RI before and during Pregnancy

**Figure 3:**
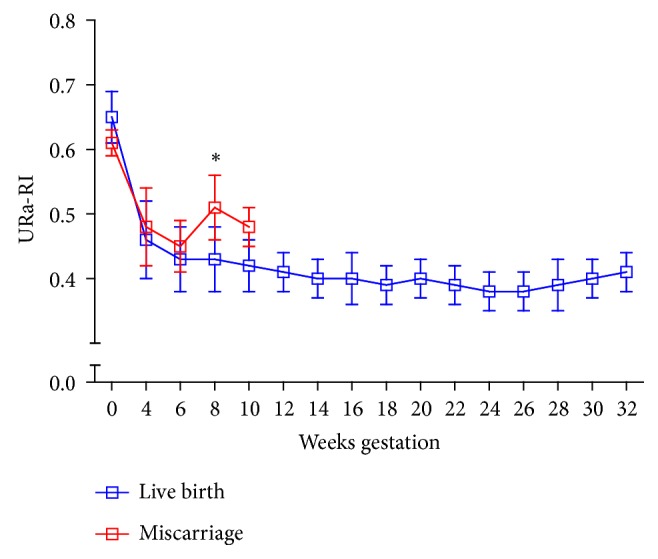
Dynamic changes of URa-RI before and during pregnancy. Women who miscarried showed a significant elevation of URa-RI at 8-week gestation compared to women who delivered a live fetus.

The changing trend of URa-RI levels before and during pregnancy is shown in Figure 3. In the majority of cases, URa-RI levels were decreased dramatically during pregnancy. URa-RI levels at 4 and 6 weeks of pregnancy were higher in women who had a miscarriage compared to those with a live birth, but the observed differences were not statistically significant. URa-RI levels at 8-week gestation were significantly higher in women who miscarried as compared with those of women who had a live birth (0.51±0.08 vs. 0.42±0.03, p<0.001). There were no differences in URa-RI levels between the two groups at 10-week gestation.

### 3.3. Receiver Operating Characteristic (ROC) Curve Analysis

**Figure 4:**
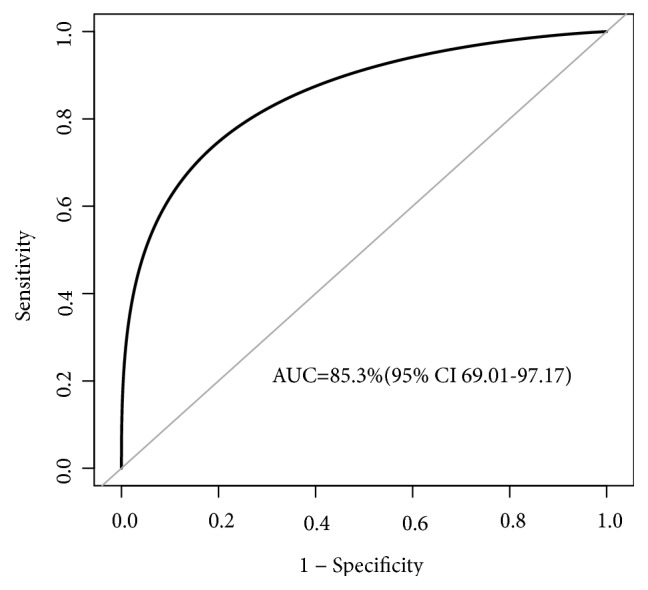
Receiver operating characteristics (ROC) curve analysis using the URa-RI level at 8-week gestation for predicting miscarriage. The URa-RI level at 8-week gestation yielded a ROC curve value of 85.29% (95% CI 69.01-97.17) in discriminating miscarriage occurrence. Bootstrap resampling times=2000. AUC=area under the curve.

ROC curve analysis was applied to further verify the discriminating power of the URa-RI level at 8-week gestation. The results demonstrated that the URa-RI level at 8-week gestation was able to effectively distinguish women who miscarried from those who had a live birth with the area under the curve (AUC) of 85.29% (95% CI 69.01-97.17) (Figure 4). ROC curve analysis for the URa-RI levels revealed a cut-off point of 0.45 as the most optimal balance of sensitivity (83.33%) and specificity (87.78%) (Table 3). Interestingly, the negative predictive value at or below this cut-off point (i.e., for ruling out miscarriage) was 97.53%.

### 3.4. Elevated URa-RI Level and Pregnancy Outcome

Out of 139 women, 23 had spontaneous miscarriages (16.5%). The association between an increased URa-RI at 8-week gestation and the risk of miscarriage was investigated. As shown in Table 4, the odds ratio (OR) for miscarriage was significantly increased as the URa-RI at 8-week gestation was increased. OR for URa-RI>=0.45 was significantly higher than the OR for URa-RI<0.45 (OR 35.91, 95% CI 6.94-185.86; P<0.0001). Additional adjustment for the confounding variables, including age, BMI, and the number of previous miscarriages, did not reduce the OR for the association between the URa-RI at 8-week gestation and the miscarriage (URa-RI≥0.45 vs. <0.45:OR49.68, 95% CI 8.01-307.95; P<0.0001). We also conducted analyses with the URa-RI at 8-week gestation as a continuous variable. Each 0.1 unit increase in the URa-RI was associated with a 15.44 increased risk of miscarriage in the nonadjusted model (95% CI, 3.75-72.14; P=0.0002). Adjusting the age, BMI, and the number of previous miscarriages did not affect the relationship.

To determine the consistency of the relationship between an increased URa-RI and risk of miscarriage, we conducted stratified analyses (Table 5). The adjusted OR for miscarriage for women less than 40 years of age was 61.9 (P=0.0001) compared with 5.71 (p=0.04) for women greater than 40 years (P interaction=0.93). The adjusted OR for women with normal body mass index (defined as BMI <23 kg/m^2^) was 20.47 (P=0.01). For persons with an elevated BMI, the OR was 18.15 (P=0.001). This disparity was consistent with random variation (P interaction=0.93). The OR for miscarriage was 29.7 in the number of previous miscarriages <5 (P=0.0002) and 6.2 in that ≥5 (P =0.09); the difference was statistically insignificant (P interaction =0.28).

## 4. Discussion

In this study, we report that a higher URa-RI assessed with the ultrasonographic scan at 8-week gestation is significantly associated with an increased risk of miscarriage in women with RPL and thrombophilia. This association was validated after adjustment for demographic and clinical information. The present study also identified a cut-off point of 0.45 for the URa-RI at 8-week gestation to predict miscarriage with the AUC of 85.29 % (95% CI 69.01-97.17).

In a normal pregnancy, placental trophoblast cells invade the inner third of the myometrium and migrate to the entire length of the maternal spiral arteries. This provides optimal delivery of oxygen and nutrients to the growing fetus [28]. In case of a failure to invade trophoblast into the uterine muscular wall, the spiral arteries retain the muscular elastic coating, which leads to an increase in RI by maintaining impedance to blood flow [29, 30]. A pathological increase in placental vascular resistance can be detected by Doppler flow studies of maternal uterine vessels, and increased RI of uterine vessels is associated with subsequent obstetrical complications during the first and second trimester [31].On the other hand, conflicting studies also have been reported, which could be due to the different methodological approaches of the studies, for instance, patient's selection criteria, definitions of abnormal Doppler ultrasound waveforms, and adverse outcome as well as Doppler insonation [32–35].

Uterine artery Doppler screening of high-risk women (such as women with URPL and thrombophilia) may predict high-risk population for adverse pregnancy outcomes [31]. Among our research candidates, URa-RI at 8-week gestation was associated with the increased risk of miscarriage independent of traditional risk factors, such as maternal age, BMI, and the number of previous miscarriages. Abnormal URa-RI in these women could potentially lead to early surveillance of pregnancy losses and interventions that might improve clinical outcomes.

In our study, the URa-RI was longitudinally monitored by ultrasonography at the periovulatory phase before pregnancy and every two weeks during pregnancy, until the occurrence of miscarriage or 32-week gestation. However, most published blood flow measurements have been constructed from cross-sectional measurements of populations with no information on subsequent pregnancy courses [36, 37]. Following individuals through repeated examinations enables to generate a longitudinal curve for RI measurements during pregnancy. In most women, URa-RI levels were sharply decreased with pregnancy. URa-RI levels at 8-week gestation were significantly higher in women who miscarried, as compared to those who had a live birth. There were no differences in URa-RI levels between the two groups on the baseline, during the 4^th^, 6^th^, and 10^th^ week of gestation.

Although maternal age, BMI, and the number of previous miscarriages are known to be risk factors for subsequent miscarriage [38, 39], these factors were not found to be correlated with the outcome of pregnancy in this study. This may be due to the on-going treatment with anticoagulant or anti-inflammatory medication and inadequate sample size. Nonetheless, these traditional risk factors were considered when we quantitatively analyzed the relationship between URa-RI and pregnancy outcome. The OR for miscarriage was significantly increased as the URa-RI at 8-week gestation was increased. The OR for URa-RI ≥0.45 (OR 35.91, 95%CI 6.94-185.86) was significantly higher than the OR for URa-RI<0.45(P<0.0001). Additional adjustment for the confounding variables (age, BMI, and the number of previous miscarriages) did not reduce the OR for the association between the URa-RI at 8-week gestation and the development of miscarriage (URa-RI ≥ 0.45 versus <0.45:OR49.68, 95%CI 8.01-307.95; P<0.0001). Overall, the adjusted OR value is much greater than 3. Therefore, increased URa-RI at 8-week gestation is considered to be an independent risk factor for miscarriage.

Many types of research in the field of RPL consisted of case-control studies, where women with RPL (cases) are compared with their unaffected counterparts (controls). Exposures are then assessed; for example, demographic factors, genetic mutations and biomarkers, and rates of exposures are compared between groups as an OR. Case-control studies are strictly observational and are easily biased if (a) cases are not well selected, (b) controls are not appropriately defined, and (c) confounders are not accounted for Schulz K [40]. Given the difficulty assessing and controlling for confounders in case-control trials, some experts in study design recommend considering OR<3 to be confounding and thus not a true association [40]. In this study, we did not choose healthy women without a history of recurrent miscarriage as a control group, rather, women from the same population having different pregnancy outcomes. This can effectively avoid selection bias. Hence the conclusion is robust.

The number of observed events is small, which limits the statistical power of this explorative study to some extent. To determine the effect of a sample size to the power, we used a power simulation model to evaluate the power of different sample size. The results were shown in Supplement [Supplementary-material supplementary-material-1]. The power was 1.0 with 130 sample size when the odds ratio was 35. The increment in the sample size would lead to a further increase in power. Nevertheless, a larger sample size may obtain a larger number of observed cases. Otherwise, the sample size of the current study is considered to be sufficient to draw a conclusion that may guide clinical practice. In conclusion, in this cohort of women with RPL and thrombophilia, the URa-RI at 8-week gestation over 0.45 is an independent risk factor for miscarriage. Further research is needed to replicate these findings in other cohorts.

## 5. Conclusions

Uterine artery Doppler interrogation has been reported to predict the second and third-trimester obstetrical complications such as preeclampsia and intrauterine growth restriction. In this study, we report that, in women with RPL and thrombophilia, increased URa-RI at 8-week gestation was associated with spontaneous abortion independent of other risk factors while they were on anticoagulation treatment. Doppler interrogation of the uterine radial artery during the first trimester may select high-risk women to miscarry during the first trimester among pregnant women with a history of RPL.

## Figures and Tables

**Table 1:** Age, BMI, number of miscarriages, and auto- and cellular immune profiles of the study group.

Characteristic	Total	Women with a live birth	Women with a miscarriage	P value
N	139	116	23	
Mean age (yr)	34.9 ± 4.5	35.14±4.33	33.52±5.12	0.12
<40	114 (82.1%)	95(81.90)	19(82.61%)	0.94
≥40	25 (18.0%)	21(18.10%)	4(17.39%)	
Mean BMI^a^	25.2 ± 4.7	24.98±4.55	26.24±5.11	0.24
<19	2(1.4%)	1(0.86%)	1(4.35%)	0.15
≥19, <25	76 (54.7%)	67(57.76%)	9(39.13%)	
≥25	61 (43.9%)	48(41.38%)	13(56.52%)	
Number of miscarriages (n)	3.7 ± 1.6	3.59±1.52	4.13±1.74	0.14
<5	107 (77.0%)	92(79.31%)	15(65.22%)	0.18
≥5	32 (23.0%)	24(20.69%)	8(34.78%)	
ANA^b^				
Negative	62 (44.6%)	54(46.55%)	8(34.78%)	0.36
Positive	77 (55.4%)	62(53.45%)	15(65.22%)	
APA^c^				
Negative	69 (49.6%)	54(46.55%)	15(65.22%)	0.36
Positive	70 (50.4%)	62(53.45%)	8(34.78%)	
CD56^+^NK cells	8.6 ± 4.7	8.38±4.70	9.56±4.92	0.21
<12%	108 (77.70%)	93(80.17%)	15(65.22)	0.17
≥12%	31 (22.30%)	23(19.83%)	8(34.78%)	
NK cell cytotoxicity				
E:T^d^=50:1	16.35±6.50	15.77±6.00	19.24±8.11	0.02
E:T=25:1	11.02±5.31	10.59±5.02	13.20±6.27	0.03
E:T=12.5:1	7.05±4.19	6.71±3.95	8.53±4.91	0.06
TNF-*α*^+^/IL-10^+^ CD4^+^ T helper cell ratio	31.6 ± 10.1	31.62 ±10.30	31.46±9.03	0.80
<30.6	66(47.48%)	59(50.86%)	7(30.43%)	0.11
≥30.6	73 (52.52%)	57(49.14%)	16(69.57%)	
IFN-*γ*^+^/IL-10^+^ CD4^+^ T helper cell ratio	15.7 ± 7.1	15.62 ±7.45	15.91±5.45	0.43
<20.5	108 (77.70%)	88(75.86%)	20(86.96%)	0.29
≥20.5	31 (22.30%)	28(24.14%)	3(13.04%)	

Data expressed as mean± SD or a number with percentage.

^a^Body-mass index (BMI) is the weight in kilograms divided by the square of the height in meters (Kg/m^2^).

^b^Antinuclear antibody (ANA). ^c^Antiphospholipid antibodies (APA).

^d^Effector to target cell ratio (E:T).

**Table 2:** Characteristics of the thrombophilia in the study group (N=139).

Thrombophilia	Positive	Negative	
Protein S deficiency	14 (10.1%)	125 (89.9%)	
Protein C deficiency	2 (1.4%)	137 (98.6%)	
PAI-1 level	33 (23.7%)	106 (76.3%)	
PAI-1 gene polymorphism	98 (70.3%)	41 (29.7%)	
HPA-1 gene polymorphism	31 (22.3%)	108 (77.7%)	
*β*-fibrinogen gene polymorphism	36 (25.9%)	103 (74.1%)	
Factor-II gene polymorphism	1 (0.7%)	138 (99.3%)	
Factor-V gene polymorphism	6 (4.3%)	133 (95.7%)	
Factor-XIII gene polymorphism	50 (36.0%)	89 (64.0%)	
MTHFR polymorphisms	111 (79.1%)	28 (20.1%)	
C677T	68 (48.9%)		
A1298C	24 (17.3%)		
A1298C/C677T	19 (13.7%)		

**Table 3:** Accuracy of uterine radial artery resistance index (URa-RI) at 8-week gestation for estimating the risk of miscarriage development.

Variable	URa-RI at 8 weeks gestation
Cut-off value	0.45
Area under ROC curve	85.29%
Sensitivity,%	83.33%
Specificity,%	87.78%
Positive predictive value,%	47.62%
Negative predictive value,%	97.53%
Positive likelihood ratio	6.82
Negative likelihood ratio	0.19

ROC, receiver operating characteristic.

Bootstrap resampling times= 2000.

**Table 4:** Risk of miscarriage associated with the elevated URa-RI at 8-week gestation.

	Live birth	Miscarriage	Nonadjusted^a^	P value	Adjust I^b^	P value	Adjust II^c^	P value
	N (%)	N (%)	(OR 95% CI)		(OR 95% CI)		(OR 95% CI)	
URa-RI at 8 wks for each 0.1 unit increase	0.42±0.03	0.51±0.08	16.44	0.0002	20.05	0.0001	19.70	0.0001
			(3.75, 72.14)		(4.36, 92.2)		(4.26, 91.1)	
<0.45	102 (87.93)	4 (17.39)	1.0		1.0		1.0	
≥0.45	14 (12.07)	19 (82.61)	35.91	<0.0001	43.97	<0.0001	49.68	<0.0001
			(6.94, 185.86)		(7.54, 256.58)		(8.01, 307.95)	

^a^Nonadjusted model.

^b^Adjust I model adjusted for age; BMI.

^c^Adjust II model adjusted for age; BMI; number of miscarriages.

**Table 5:** Odds ratios with 95% confidence intervals for pregnancy outcome per 0.1 unit increase of URa-RI at 8-week gestation in subgroups of age, BMI, and number of miscarriages.

	No. of events	OR (95% CI)	P value	P for interaction
Age				
<40	136	61.9 (7.55-507.7)	0.0001	0.0718
≥40	42	5.71 (1.22-26.62)	0.0267	
BMI				
<25	100	20.47 (2.08-201.4)	0.0096	0.9346
≥25	78	18.15 (3.19-103.21)	0.0011	
Number of miscarriages				
<5	147	29.7 (5.1-173.8)	0.0002	0.2753
≥5	31	6.2 (0.8-48.5)	0.0909	

## Data Availability

The ultrasound and clinical characteristics data used to support the findings of this study are restricted by the IRB at Rosalind Franklin University of Medicine and Science in order to protect patient privacy. Data are available from Joanne Kwak-Kim, MD (joanne.kwakkim@rosalindfranklin.edu), for researchers who meet the criteria for access to confidential data.

## Supplementary Materials

Supplementary Materials Supplement Figure 1: the power of different sample size was shown when URa-RI was used as a continuous (A) and a binary categorical variable (B), respectively.
