# Supplementary material for: Uterine Radial Artery Resistance Index Predicts Reproductive Outcome in Women with Recurrent Pregnancy Losses and Thrombophilia
Source: Biomed Res Int. 2019 Jun 20;2019:8787010. doi: 10.1155/2019/8787010 (PMC6724444; doi:10.1155/2019/8787010)
Supplement: Supplementary Materials — Supplement Figure 1: the power of different sample size was shown when URa-RI was used as a continuous (A) and a binary categorical variable (B), respectively. [file 8787010.f1.pdf]

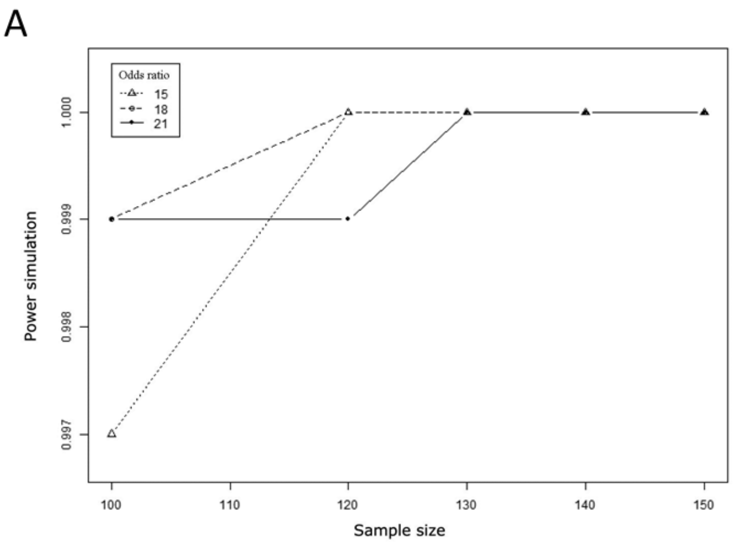

The power of different sample size with URa-RI as a continuous variable.

	OR=15	OR=18	OR=21
N= 100	0.997	0.999	0.999
N= 120	1	1	0.999
N= 130	1	1	1
N= 140	1	1	1
N= 150	1	1	1

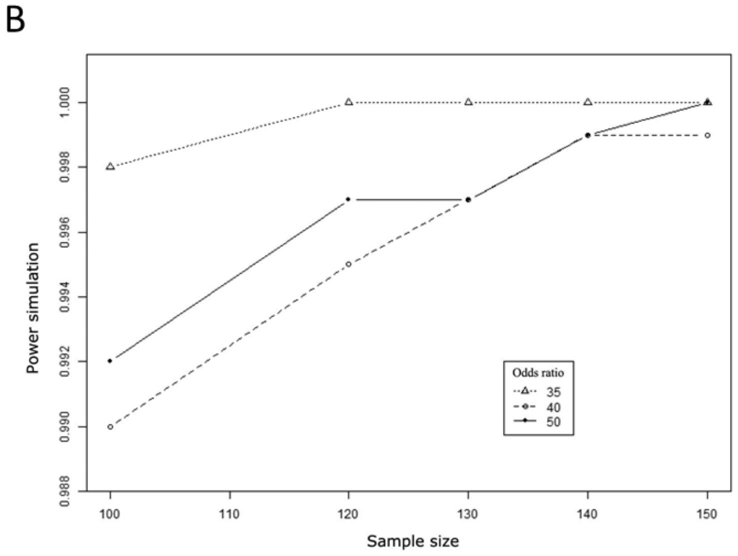

The power of different sample size with URa-RI as a binary categorical variable.

	OR= 35	OR =40	OR= 50
N= 100	0.998	0.990	0.992
N= 120	1	0.995	0.997
N= 130	1	0.997	0.997
N= 140	1	0.999	0.999
N= 150	1	0.999	1

Supplement figure 1. The power of different sample size was shown when URa-RI was used as a continuous (A) and a binary categorical variable (B), respectively.
